# Axonal distribution of mitochondria maintains neuronal autophagy during aging via eIF2β

**DOI:** 10.1101/2024.01.20.576435

**Published:** 2024-01-20

**Authors:** Kanako Shinno, Yuri Miura, Koichi M. Iijima, Emiko Suzuki, Kanae Ando

**Affiliations:** 1. Department of Biological Sciences, Graduate School of Science, Tokyo Metropolitan University, Hachioji, Tokyo, 192-0397, Japan; 2. Research Team for Mechanism of Aging, Tokyo Metropolitan Institute for Geriatrics and Gerontology, Itabashi, Tokyo, 173-0015, Japan; 3. Department of Alzheimer’s Disease Research, National Center for Geriatrics and Gerontology, Obu, Aichi, 474-8511, Japan; 4. Department of Experimental Gerontology, Graduate School of Pharmaceutical Sciences, Nagoya City University, Nagoya, Aichi, 467-8603, Japan; 5. Gene Network Laboratory, National Institute of Genetics and Department of Genetics, SOKENDAI, Mishima, Shizuoka, 411-8540, Japan; 6. Department of Biological Sciences, School of Science, Tokyo Metropolitan University, Hachioji, Tokyo, 192-0397, Japan

**Keywords:** Mitochondria, axonal transport, aging, neuronal proteostasis, protein aggregation, autophagy, Eukaryotic Initiation Factor 2β (eIF2β), *Drosophila*, neurodegeneration, proteome

## Abstract

Neuronal aging and neurodegenerative diseases are accompanied by proteostasis collapse, while cellular factors that trigger it are not identified. Impaired mitochondrial transport in the axon is another feature of aging and neurodegenerative diseases. Using *Drosophila*, we found that genetic depletion of axonal mitochondria causes dysregulation of translation and protein degradation. Axons with mitochondrial depletion showed abnormal protein accumulation, and autophagic defects. Lowering neuronal ATP levels by blocking glycolysis did not reduce autophagy, suggesting that autophagic defects are associated with mitochondrial distribution. We found eIF2β was upregulated by depletion of axonal mitochondria via proteome analysis. Phosphorylation of eIF2α, another subunit of eIF2, was lowered, and global translation was suppressed. Neuronal overexpression of *eIF2β* phenocopied the autophagic defects and neuronal dysfunctions, and lowering *eIF2β* expression rescued those perturbations caused by depletion of axonal mitochondria. These results indicate the mitochondria-eIF2β axis maintains proteostasis in the axon, of which disruption may underly the onset and progression of age-related neurodegenerative diseases.

## Introduction

Neurons have a morphologically complex architecture composed of microcompartments and require tight regulation of the abundance of proteins and organelles spatially and temporally^1^. Such control of protein amounts, or proteostasis, is essential for neuronal functions^2^ and is achieved through the orchestration of protein expression, folding, trafficking, and degradation controlled by intrinsic and environmental signals^3^. Translation is initiated by the eukaryotic initiation factor 2 (eIF2) complex^4^. eIF2, a heterotrimer of α, β, and γ subunits, transports Met-tRNA to the ribosome in a GTP-dependent manner^5^. Under stressed conditions, phosphorylation of eIF2α attenuates global translation and initiates translation of mRNAs related to the integrated stress response (ISR)^6^. As for protein degradation, the autophagosome and proteasome are major systems that maintain proteostasis^7^. The proteasome degrade unnecessary proteins followed by regulated ubiquitination processes^8^, and autophagy removes damaged or harmful components, including large protein aggregates and organelles, through catabolism (selective autophagy)^9^. In addition to autophagy induced by acute stressors, a basal level of selective autophagy mediates the global turnover of damaged proteins^10^.

Such constitutive autophagy decreases during aging, which may underlie declines in the structural and functional integrity of neurons^11^. Decreased protein degradation and accumulation of abnormal proteins also contribute to increased risks of neurodegenerative diseases. Age-related neurodegenerative diseases such as Alzheimer’s disease and Parkinson’s disease are often associated with the accumulation of misfolded proteins such as amyloid-β, tau, and α-synuclein^12^. Enhancement of autophagy mitigates age-related dysfunctions and neurodegeneration caused by proteotoxic stress^13^. However, it is not fully understood how aging disrupts the regulation of this constitutive autophagy.

Neurons are also highly energy-demanding. At nerve terminals, action potentials trigger the release of neurotransmitters via exocytosis of synaptic vesicles, which requires a constant supply of ATP and calcium buffering^14^. Such neuronal activity relies on mitochondrial functions^15^, and mitochondria are actively transported from their major sites of biogenesis in soma to axons^16^. However, the axonal transport of mitochondria declines during aging^17 18 19^. Reduced axonal transport of mitochondria is thought to contribute to age-related declines in neuronal functions^17 19 20 21^. The number of functional mitochondria in synapses is reduced in the brains of patients suffering from age-related neurodegenerative diseases such as Alzheimer’s disease^22^, and mutations in genes involved in mitochondrial dynamics are linked to neurodegenerative diseases^23^. Mislocalization of mitochondria is sufficient to cause age-dependent neurodegeneration in *Drosophila* and mice^24,25^, indicating that the proper distribution of mitochondria is essential to maintain neuronal functions. Thus, depletion of functional mitochondria from axons and proteostasis collapse are common features of aging and neurodegenerative diseases.

Mitochondrial transport is regulated by a series of molecular adaptors that mediate the attachment of mitochondria to molecular motors^16^. In *Drosophila*, mitochondrial transport is mediated by milton and Miro, which attaches mitochondria to microtubules via kinesin heavy chain^26,27^. In the absence of milton or Miro, synaptic terminals and axons lack mitochondria, although mitochondria are numerous in the neuronal cell body^28^ . We previously reported that RNAi-mediated knock-down of *milton* or *Miro* in neurons causes reduction in axonal mitochondria, age-dependent locomotor defects^29^, and age-dependent neurodegeneration in neuropile area starting around 30 days after eclosion (day-old)^24^, and enhances axon degeneration caused by human tau proteins^24^. These results suggest that flies with neuronal knockdown of *milton* can be used as a model to analyze the effect of depletion of axonal mitochondria during aging. In this study, we investigated a causal relationship between mitochondrial distribution and neuronal proteostasis by using neuronal knockdown of *milton*. We found that depletion of axonal mitochondria reduced autophagy and increased accumulation of aggregated proteins in the axon prior to neurodegeneration. Proteome analysis and follow-up biochemical analyses revealed that neuronal knockdown of *milton* increased eIF2β levels. In addition, phosphorylation of eIF2α was lowered in the axon, and global translation was suppressed. Overexpression of *eIF2β* was sufficient to decrease autophagy and induce neuronal dysfunction, and genetic suppression of *eIF2β* restored autophagy and improved neuronal function in the *milton* knockdown background. These findings suggest that loss of axonal mitochondria and elevated levels of eIF2β mediate proteostasis collapse and neuronal dysfunction during aging.

## Results

### Depletion of axonal mitochondria by knockdown of *milton* or *Miro* causes protein accumulation in the axon

**Figure 1.**
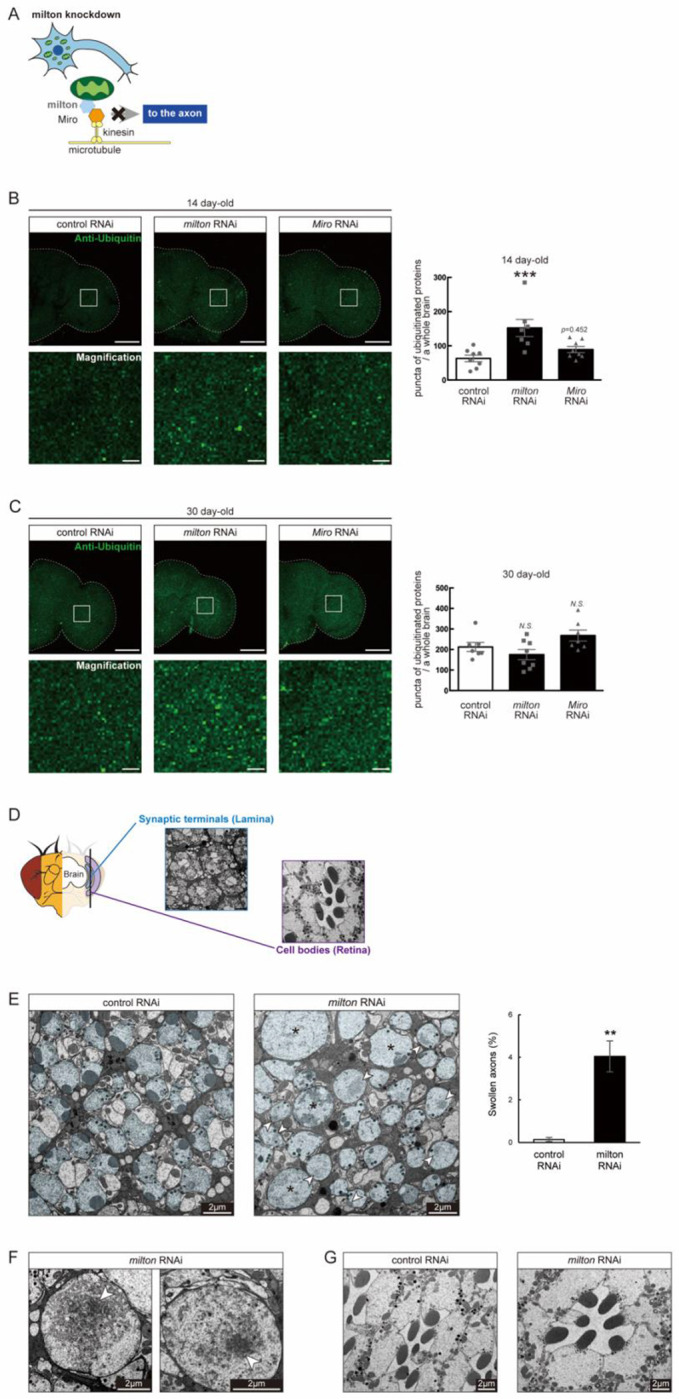
Knockdown of *milton* or *Miro* causes protein accumulationin the axon (A) Schematic representation of the mitochondrial transport machinery. (B, C) Ubiquitinated proteins in brains with neuronal knockdown of *milton* or *Miro*. Brains dissected at 14 day-old (B) or 30 day-old (C) were immunostained with an antibody against ubiquitinated proteins. Firefly *luciferase* RNAi was used as a control. Representative images (left) and quantitation of the number of ubiquitin-positive puncta (right) are shown. Scale bars of hemibrains, 100 μm, Scale bars of magnifications, 10 μm. Means ± SE, n = 8. *N.S.*, *p* > 0.05; ***p < 0.005 (one-way analysis of variance (ANOVA) followed by Tukey’s honestly significant difference (HSD) *post hoc* test). (D) Cross sections in the lamina and in the retina were used to analyze the ultrastructure of synapses and cell bodies, respectively. milton RNAi was expressed in the retina and neurons via a combination of GAL4 drivers, a pan-retinal gmr-GAL4 and pan-neuronal elav-GAL4. (E, F) Transmission electron micrographs of presynaptic terminals of photoreceptor neurons of control and *milton* knockdown flies. Photoreceptor axons are highlighted in blue. Swollen axons (asterisks in (E)) are observed in *milton* knockdown neurons. Scale bars, 2 μm. Representative images and quantitation are shown. In total, 918–1118 cells from cross-sections of the lamina from each half of the head were analyzed. Mean ± SE, n = 3. **p < 0.001 (Student’s *t*-test). (F) Dense materials (arrowheads) in synaptic terminals of *milton* knockdown neurons. Scale bars, 2 μm. (G) Cell bodies of photoreceptor neurons of control and *milton* knockdown flies. Scale bars, 2 μm. Flies were 27 day-old.

In *Drosophila*, mitochondrial transport is mediated by milton and Miro, which attaches mitochondria to microtubules via kinesin heavy chain^26,27^ (Figure 1A). It has been reported that expression of *milton* RNAi in neurons via pan-neuronal elav-GAL4 driver reduced milton protein levels in *Drosophila* head lysate to 40% and mito-GFP signals in axons to 50%^29^.

To test how loss of axonal mitochondria affects proteostasis in neurons, we first examined the accumulation of ubiquitinated proteins. At 14 day-old, more ubiquitinated proteins were deposited in the brains of *milton* knockdown flies than in those of age-matched control flies (Figure 1B, *p* < 0.005 between control RNAi and *milton* RNAi). Knockdown of *Miro*, a partner of *milton*, also resulted in more ubiquitin-positive puncta, but the difference was not significant (Figure 1B, *p* = 0.452 between control RNAi and *Miro* RNAi). These data suggest that the depletion of axonal mitochondria leads to accumulation of ubiquitinated proteins before neurodegeneration occurs.

It has been reported that ubiquitinated proteins accumulate with aging^30^; thus, we analyzed the accumulation of ubiquitinated proteins in aged brains (30 day-old) with *milton* knockdown. The number of puncta of ubiquitinated proteins did not significantly differ between control and *milton* knockdown flies or between control and *Miro* knockdown flies (Figure 1C, *p* > 0.05). Together with the effect of *milton* knockdown in 14-day old flies, this result suggests that depletion of axonal mitochondria accelerates the onset of age-related accumulation of ubiquitinated proteins.”

We examined the ultrastructure of synaptic terminals and cell bodies in photoreceptor neurons with *milton* knockdown by transmission electron microscopy in 27 day-old flies (Figure 1D). As previously reported^24^, axonal swelling was observed in these neurons (Figure 1E, asterisks). Some swollen axons in *milton* knockdown neurons contained dense materials, indicating abnormal protein accumulation in the axon (Figure 1F, arrowheads). In contrast, dense materials were not observed in cell bodies in the *milton* knockdown retina (Figure 1G). These results indicate that the depletion of axonal mitochondria induces protein accumulation in the axon.

### Depletion of axonal mitochondria impairs protein degradation pathways

**Figure 2.**
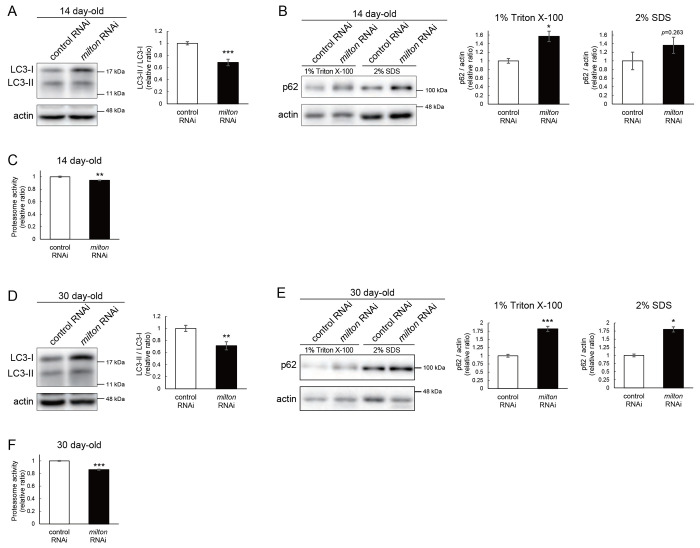
*milton* knockdown impairs protein degradation pathways (A, B) Western blotting of head extracts of control and *milton* knockdown flies with antibodies against LC3 (A) and Ref2P, the fly homolog of mammalian p62 (B). For assessment of p62 levels, heads were extracted with 1% Triton X-100 or 2% SDS (B). Flies were 14 day-old. Representative blots (left) and quantitation (right) are shown. Actin was used as a loading control. Means ± SE, n = 6 (LC3), n = 3 (p62). (C) Proteasome activity in head extracts of control and *milton* knockdown flies was measured by hydrolysis of Suc-LLVY-AMC (chymotrypsin-like activity) at 14 day-old. Means ± SE, n =3. (D, E) Western blotting of head extracts of 30 day-old control and *milton* knockdown flies. Blotting was performed with anti-LC3 (D) and anti-p62 (E) antibodies. Representative blots (left) and quantitation (right) are shown. Actin was used as a loading control. Means ± SE, n = 6 (LC3), n = 3 (p62). (F) Proteasome activity in head extracts of 30-day-old control and *milton* knockdown flies was measured by hydrolysis of Suc-LLVY-AMC (chymotrypsin-like activity). Means ± SE, n = 3. *N.S.*, *p* > 0.05; *p < 0.05; **p < 0.001; ***p < 0.005 (Student’s *t*-test).

Since abnormal proteins were accumulated in *milton* knockdown brains, we next examined if protein degradation pathways were suppressed. We analyzed autophagy via western blotting of the autophagy markers LC3 and p62^31^. During autophagy progression, LC3 is conjugated with phosphatidylethanolamine to form LC3-II, which localizes to isolation membranes and autophagosomes. The LC3-II/LC3-I ratio was lower in the head lysate of *milton* knockdown flies than in that of control flies (Figure 2A). We also analyzed an autophagy substrate p62. Brain lysates were sequentially extracted using detergents with different stringencies (1% Triton X-100 and 2% SDS), and p62 was detected by western blotting^32^. The p62 level was increased in brains of 14 day-old *milton* knockdown flies (Figure 2B), indicating that autophagy is decreased in these brains. The increase in the p62 level was more prominent in the Triton X-100-soluble fraction than in the SDS-soluble fraction (Figure 2B), suggesting that depletion of axonal mitochondria impairs the degradation of less-aggregated proteins. We also analyzed proteasome activity. At 14 day-old, 20S proteasome activity was significantly decreased in brains with neuronal knockdown of *milton* (Figure 2C, *p* < 0.005). Autophagy was also reduced in the brains of *milton* knockdown flies at 30 day-old (Figure 2D). At this age, milton knockdown increased p62 in both fractions significantly (Figure 2E). Proteasome activities were also decreased in *milton* knockdown flies at 30 day-old (Figure 2F). These results indicate that depletion of axonal mitochondria impairs protein degradation pathways.

### ATP deprivation does not impair autophagy

**Figure 3.**
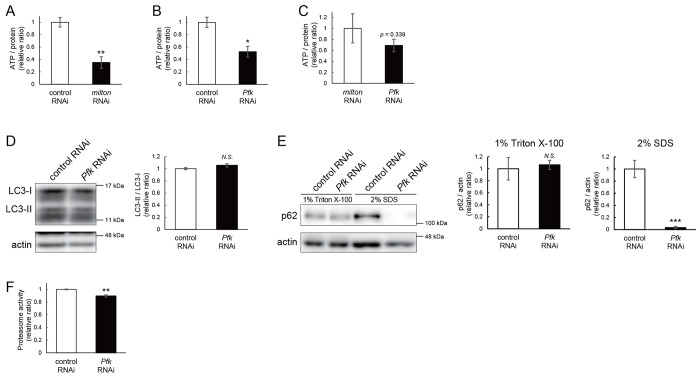
ATP deprivation does not impair autophagy (A-C) ATP levels in brain extracts of control and *milton* knockdown flies (A) and control and *Pfk* knockdown flies (B), and comparison of the effects of *milton* knockdown and *Pfk* knockdown on ATP levels (C). Flies were 14 day-old. Means ± SE, n = 3. (D, E) Western blotting of head extracts of flies with neuronal expression of control or *Pfk* RNAi. Blotting was performed with anti-LC3 (D) and anti-p62 (E) antibodies. For assessment of p62 levels, heads were extracted with 1% Triton X-100 or 2% SDS. Representative blots (left) and quantitation (right) are shown. Actin was used as a loading control. Means ± SE, n = 6 (LC3), n = 3 (p62). (F) Proteasome activity in head lysates of flies with neuronal expression of control or *Pfk* RNAi was measured by hydrolysis of Suc-LLVY-AMC (chymotrypsin-like activity). Means ± SE, n = 3. *N.S.*, *p* > 0.05; *p < 0.05; **p < 0.001; ***p < 0.005 (Student’s *t*-test). Flies were at 14 day-old.

*milton* knockdown downregulates ATP in the axon^33^. To examine whether the disruption of protein degradation pathways by *milton* knockdown is due to ATP deprivation, we investigated the effects of knocking down phosphofructokinase (*Pfk*), a rate-limiting enzyme in glycolysis, on protein degradation pathways. Neuronal knockdown of *Pfk* was reported to lower ATP levels in brain neurons^33^. *Pfk* knockdown and *milton* knockdown decreased ATP to similar levels (Figure 3A–C). However, in contrast with *milton* knockdown, *Pfk* knockdown did not affect the LC3-II/LC3-I ratio (Figure 3D) and decreased p62 level (Figure 3E), suggesting that autophagy is promoted. On the other hand, proteasome activity was decreased by *Pfk* knockdown (Figure 3F). These results suggest that the downregulation of axonal ATP upon depletion of axonal mitochondria decreases proteasome activity, but not autophagy.

### Proteome analysis suggests that depletion of axonal mitochondria causes disruption of autophagy and premature aging

**Figure 4.**
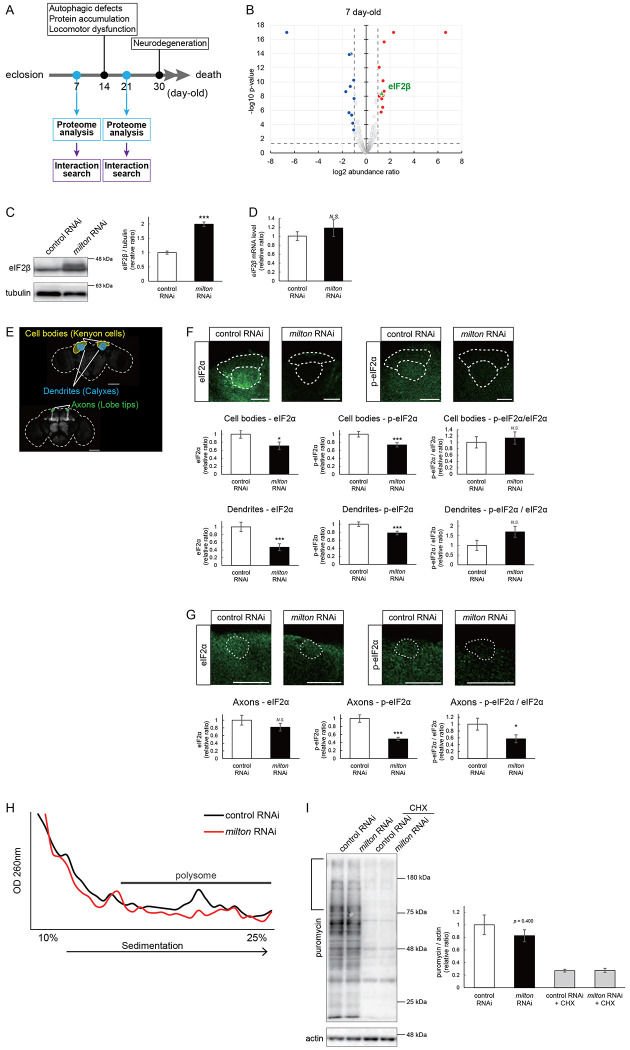
*milton* knockdown upregulates eIF2β and decreases phosphorylation of eIF2α and translation (A) Age of proteome analysis and phenotypes of *milton* knockdown flies. (B) A scatter plot of the log_2_ abundance ratio (x-axis) against the −log_10_ p-value (y-axis) of proteins at 7 day-old. (C) Western blotting of head extracts of flies expressing control or *milton* RNAi in neurons with an anti-eIF2β antibody. Flies were 14 day-old. Representative blots (left) and quantitation (right) are shown. Tubulin was used as a loading control. Means ± SE, n = 6. (D) *eIF2β* mRNA levels quantified by qRT-PCR. Means ± SE, n = 4. (E) A schematic representation of the axon (Lobe tips), the cell body region (Kenyon cells), and dendritic region (Calyxes) in the fly brain. Scale bars, 100μm. (F, G) Immunostaining with anti-eIF2α and anti-p-eIF2α antibodies. The mushroom body was identified by expression of mito-GFP. Scale bars, 20μm. The signal intensities of eIF2α and p-eIF2α in axons, dendrites, and cell bodies were quantified and are shown as ratios relative to the control. Means ± SE, n =12. (H) Representative polysome traces of head lysates of control and *milton* knockdown flies. (I) Western blotting of head lysates of control and *milton* knockdown flies fed puromycin alone or puromycin and cycloheximide (CHX) with an anti-puromycin antibody. Flies were 14 day-old. Actin was used as a loading control. Representative blots (left) and quantitation (right) are shown. Means ± SE, n = 3. *N.S.*, *p* > 0.05; *p < 0.05; ***p < 0.005 (Student’s *t*-test).

To identify the pathways that mediate the decrease in autophagy in *milton* knockdown brains, we performed proteome analysis to systematically detect differentially expressed proteins upon neuronal knockdown of *milton*. We analyzed flies at 7 and 21 day-old, the age before autophagic defects are detected and the age just before the onset of neurodegeneration, respectively. (Figure 4A). 1039 proteins were detected by liquid chromatography-tandem mass spectrometry (LC-MS/MS). Expression of 36 proteins was significantly increased (22 proteins) or decreased (14 proteins) by *milton* knockdown at 7 day-old (Tables 1 and S1). At 21 day-old, the expression of 41 proteins (31 upregulated and ten downregulated proteins) was significantly altered in *milton* knockdown flies compared with control flies (Tables 2 and S1). The “Interaction search” algorithm using KeyMolnet showed that proteins whose expression was significantly altered in the brains of *milton* knockdown flies at both 7 and 21 day-old were closely associated with the autophagic pathway (Tables 3 and 4, respectively). Proteins involved in pathways characteristic of aging, such as the immune response (Transcriptional regulation by STAT), cancer (Transcriptional regulation by SMAD, Transcriptional regulation by myc), longevity (Transcriptional regulation by FOXO, Sirtuin signaling pathway), and stress responses (HSP90 signaling pathway, MAPK signaling pathway)^34,35^, were enriched in the proteome profiles of *milton* knockdown flies compared with those of control flies (Tables 3 and 4), suggesting that depletion of axonal mitochondria accelerates aging in the brain.

### Depletion of axonal mitochondria upregulates eIF2β and decreases phosphorylation of eIF2α and translation

Differentially expressed proteins in 7 day-old flies may reflect alterations that are causal for autophagic defects. We noticed that the expression level of eIF2β was 2.465-fold higher in the brains of *milton* knockdown flies than in those of control flies (Figure 4B). Upregulation of eIF2β in the brains of *milton* knockdown flies was confirmed by western blotting. *milton* knockdown upregulated eIF2β protein levels more than twice (Figure 4C), but did not change the level of *eIF2β* mRNA (Figure 4D). eIF2β is a subunit of the eukaryotic initiation factor 2 (eIF2) complex, which is critical for translation initiation and the integrated stress response (ISR)^4^. eIF2 is a heterotrimer of α, β, and γ subunits, and during the ISR, eIF2α is phosphorylated^6^. To characterize the effect of *milton* knockdown on the eIF2α, we analyzed the levels of eIF2α and p-eIF2α in the brain. Western blotting of brain lysates showed that *milton* knockdown reduced eIF2α levels, but p-eIF2α levels were not significantly affected (Figure S1). To analyze local changes of eIF2α and p-eIF2α, we carried out immunostaining. We focused on the mushroom body, where axons, dendrites, and cell bodies can be easily identified (Figure 4E). Both eIF2α and p-eIF2α were downregulated in the cell body (Kenyon cells) and dendritic (Calyxes) regions of the brains of *milton* knockdown flies (Figure 4F). In axons (lobe tips), *milton* knockdown did not downregulate eIF2α (Figure 4G, *p* = 0.271) but significantly downregulated p-eIF2α (Figure 4G). The ratio of p-eIF2α to eIF2α was lower in the axon but not in the soma or dendritic region.

**Figure 5.**
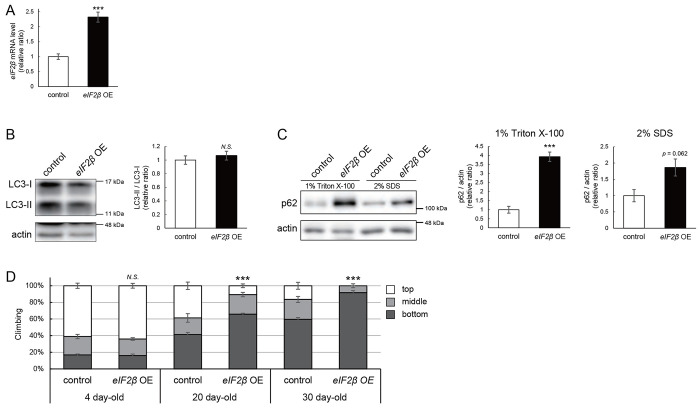
eIF2β upregulation impairs autophagy and decreases locomotor function (A) *eIF2β* mRNA levels in head extracts of flies with UAS-*eIF2β* driven by elav-Gal4 (*eIF2β* OE) or UAS-GFP driven by elav-Gal4 (control) were quantified by qRT-PCR. Flies were 2–3 day-old. Means ± SE, n=4. (B-C) Western blotting of head extracts with anti-LC3 (B) and anti-p62 (C) antibodies. Flies were 14 day-old. Representative blots (left) and quantitation (right) are shown. Tubulin and actin were used as loading controls. Means ± SE, n = 3 (p62), n = 5 (LC3). (D) Climbing assay revealed early-onset age-dependent locomotor defects in *eIF2β*-overexpressing flies. Means ± SE, n = 5. *N.S.*, *p* > 0.05; ***p < 0.005 (Student’s *t*-test).

Downregulation of p-eIF2α alters global translation^36^. We performed polysome gradient centrifugation to examine the level of ribosome binding to mRNA. In the brains of *milton* knockdown flies, the level of mRNAs associated with polysomes was reduced (Figure 5H), as expected upon downregulation of p-eIF2α. We also compared the level of translation between the brains of control and *milton* knockdown flies by assessing the incorporation of puromycin (Figure 4I). Puromycin incorporation was lower in the brains of *milton* knockdown flies than in those of control flies (Figure 4I, indicated by a bracket). These data indicate that depletion of axonal mitochondria disrupts eIF2 functions in axons and suppresses global translation.

### eIF2β upregulation reduces the level of p-eIF2α, impairs autophagy, and decreases locomotor function

We were motivated to ask if eIF2β upregulation mediates autophagic defects caused by milton knockdown. If so, neuronal overexpression of *eIF2β* would also induce autophagy impairment. Neuronal overexpression of *eIF2β* did not affect the LC3-II/LC3-I ratio (Figure 5A and B). However, overexpression of *eIF2β* significantly increased the p62 level in the Triton X-100-soluble fraction (Figure 5C, 4-fold vs. control, *p* < 0.005 (1% PBST)). The p62 level in the SDS-soluble fraction was not significantly affected (Figure 5C, 2-fold vs. control, *p* = 0.062 (2% SDS)), as observed in brains of *milton* knockdown flies (Figure 2B). Neuronal overexpression of *eIF2β* did not affect the eIF2α level but significantly decreased the p-eIF2α level (Figure S2).

Depletion of axonal mitochondria causes an age-dependent decline in locomotor function^24^, which we also observed in flies with neuronal overexpression of *eIF2β* (Figure 5D). Locomotor functions were significantly impaired in those flies at 20 day-old and worsened further during aging (Figure 5D, compare 4, 20, and 30 day-old).

These data indicate that upregulation of eIF2β in neurons phenocopies depletion of axonal mitochondria, including suppression of autophagy, and age-dependent locomotor dysfunction, suggesting that upregulation of eIF2β mediates these phenotypes downstream of loss of axonal mitochondria.

### Lowering *eIF2β* rescues autophagic impairment and locomotor dysfunction induced by *milton* knockdown

**Figure 6.**
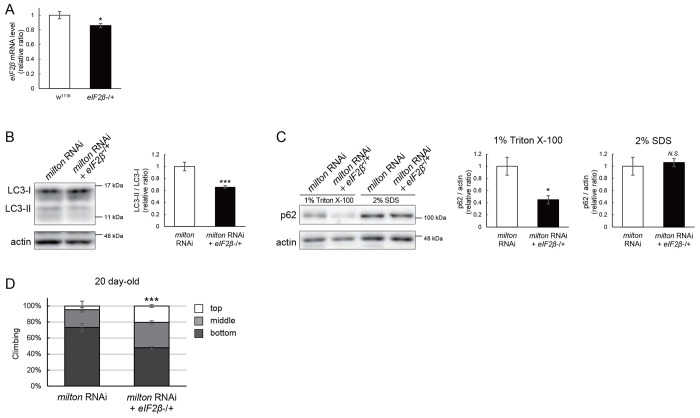
Lowering *eIF2β* rescues autophagic impairment and locomotor dysfunction induced by *milton* knockdown (A) *eIF2β* mRNA levels with one disrupted copy of the *eIF2β* gene (*eIF2β*SAstopDsRed/+ (*eIF2β* −/+)). Head extracts of flies 2–3 day-old were analyzed by qRT-PCR. Means ± SE, n = 3. (B, C) Western blotting of head extracts of flies with neuronal expression of *milton* RNAi with or without *eIF2β* heterozygosity with anti-LC3 (B) and anti-p62 (C) antibodies. Flies were 14 day-old. Representative blots (left) and quantitation (right) are shown. Actin was used as a loading control. Means ± SE, n = 5 (LC3), n = 3 (p62). (D) The climbing ability of 20 day-old flies expressing *milton* RNAi with or without *eIF2β* heterozygosity. Means ± SE, n = 15. *N.S.*, *p* > 0.05; *p < 0.05; ***p < 0.005 (Student’s *t*-test).

Finally, we investigated whether suppression of eIF2β rescues autophagy impairment and locomotor dysfunction caused by neuronal knockdown of *milton*. Null mutants and flies with RNAi-mediated knockdown of *eIF2β* in neurons did not survive. Flies lacking one copy of the *eIF2β* gene survived without any gross abnormality, and the level of *eIF2β* mRNA in these flies was about 80% of that in control flies (Figure 6A). *eIF2β* heterozygosity did not affect the eIF2α and p-eIF2α levels (Figure S3 A and B). However, the LC3-II/LC3-I ratio was significantly lower in *milton* knockdown flies with *eIF2β* heterozygosity than in flies with *milton* knockdown alone (Figure 6B). Reduction of *eIF2β* expression decreased the p62 level in the Triton X-100-soluble fraction, but not the SDS-soluble fraction, in the brains of *milton* knockdown flies (Figure 6C). Given that *milton* knockdown increased the p62 level in the Triton X-100-soluble fraction but not in the SDS-soluble fraction (Figure 2B), these results indicate that suppression of *eIF2β* ameliorates the impairment of autophagy caused by *milton* knockdown.

*eIF2β* heterozygosity also rescued locomotor dysfunction induced by *milton* knockdown. *milton* knockdown flies with *eIF2β* heterozygosity exhibited better locomotor function than *milton* knockdown alone (Figure 6D). The *milton* mRNA level was not increased in these flies, indicating that the rescue effect in the *eIF2β* heterozygous background was not mediated by an increased *milton* mRNA level (Figure S3D). These data suggest that eIF2β upregulation mediates autophagy impairment and locomotor dysfunction caused by depletion of axonal mitochondria.

## Discussion

**Figure 7.**
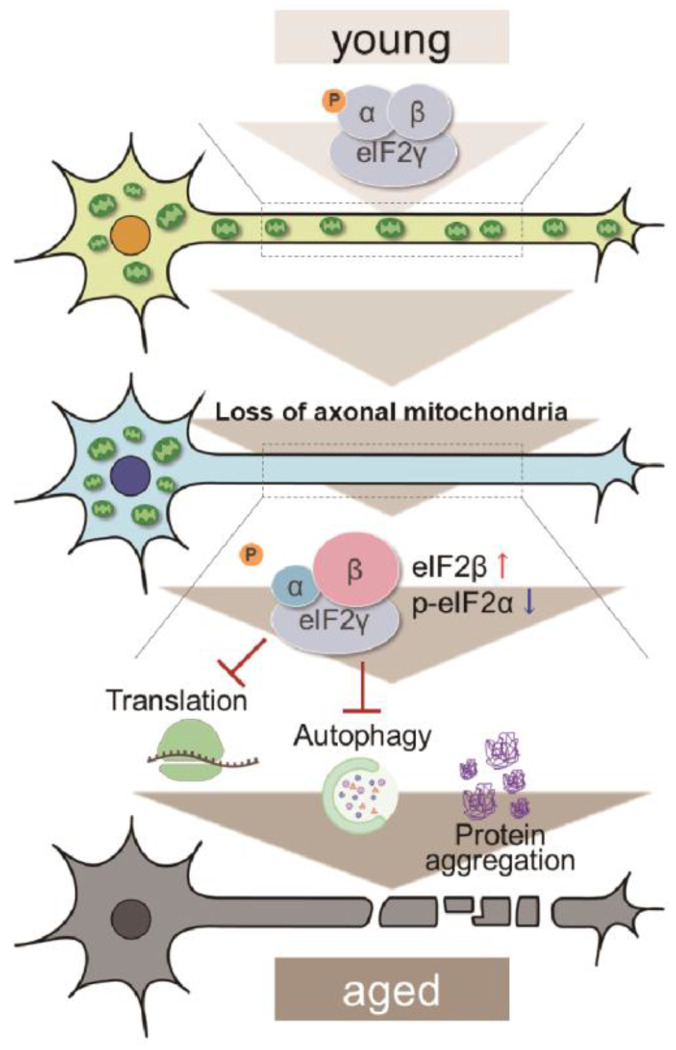
A schematic representation of the mitochondria-eIF2β axis in the axon to maintain neuronal proteostasis during aging

Proteostasis perturbations trigger the formation of pathological aggregates and increase the risks of neurodegenerative diseases during aging. By using neuronal knockdown as a tool to deplete mitochondria from the axon, we provide evidence that loss of axonal mitochondria drives age-related proteostasis collapse via eIF2β (Figure 7). We observed declines in autophagy-mediated degradation of less-aggregated proteins and proteasome activity in *milton* knockdown flies (Figure 2). Accumulation of ubiquitinated proteins and changes in age-related pathways started prematurely in *milton* knockdown flies (Figure 1 and 5). *milton* knockdown upregulated eIF2β and downregulated eIF2α phosphorylation (Figure 4). Overexpression of *eIF2β* phenocopied the effects of *milton* knockdown, including reduced autophagy and accelerated age-related locomotor defects (Figure 5). Furthermore, lowering *eIF2β* levels suppressed the impairment of autophagy and locomotor dysfunction induced by *milton* knockdown (Figure 6). Our results suggest that mitochondrial distribution and eIF2β are part of the mechanisms constituting proteostasis. *milton* knockdown causes loss of mitochondria in the axon and accumulation of mitochondria in the soma. Thus, accumulation of mitochondria may also have detrimental effects. However, degeneration induced by milton knockdown is prominent in the axon and not detected in the cell body^24^. Furthermore, abnormal protein accumulation was observed in the axon (Figure 1F) and p-eIF2α/eIF2α was decreased in the neurites but not in the soma (Figure 4F and G). These results suggest that proteostasis defects observed in these flies are caused by depletion of mitochondria in the axon rather than accumulation of mitochondria in the soma.

Our results revealed that eIF2β regulates proteostasis via autophagy and neuronal function during aging. eIF2β is a component of eIF2, which meditates translational regulation and ISR initiation. When ISR is activated, eIF2α phosphorylation suppresses global translation and induces translation of ATF4, which mediates transcription of autophagy-related genes^37,38^. In milton knockdown neurons, however, both eIF2α and global translation were reduced. Increased levels of eIF2β may disrupt eIF2 complex and its functions. We found that eIF2β upregulation reduced the levels of p-eIF2α (Figure S2), indicating that eIF2β negatively regulates eIF2α. Thus, impairment of autophagy caused by depletion of axonal mitochondria may be due to impairment of ISR. It is also possible that eIF2β mediates autophagy defects via mechanisms independent of ISR, since eIF2β has functions independent of eIF2^39,40^. For example, suppression of *eIF2β* has been reported to slow down cancer cell growth^39^. In developing neurons, eIF2β can directly interact with the translational repressor Kra to regulate midline axon guidance^40^. However, to our knowledge, the roles of eIF2β in aging have not been reported. Our results revealed a novel function of eIF2β to maintain proteostasis during aging.

How depletion of axonal mitochondria upregulates eIF2β is currently under investigation. A major mitochondrial function is ATP production, and depletion of axonal mitochondria downregulates ATP in axons^33^. However, we found that ATP deprivation does not always suppress autophagy (Figure 3), suggesting it is unlikely to be involved in the mechanisms that induce eIF2β upregulation. Mitochondria also serve as signaling hubs for translation and protein degradation. Mitochondrial proteins are regulated by co-translational protein quality control, and mitochondrial damage induces translational stalling of mitochondrial outer membrane-associated *complex-I 30 kD subunit* (*C-I30*) mRNA^41^. Additionally, the mitochondrial outer membrane ubiquitin ligase MITOL (also known as MARCHF5) ubiquitinates and regulates not only mitochondrial proteins such as Mfn2^42^ but also microtubule-associated^43^ and endoplasmic reticulum^44^ proteins. These findings indicate that mitochondria serve as local signaling centers for proteostasis maintenance and eIF2β levels may also be regulated by mechanisms related to mitochondria.

In conclusion, our results suggest that axonal mitochondria and eIF2β form an axis to maintain constitutive autophagy. Suppression of *eIF2β* rescued autophagic defects and neuronal dysfunction upon loss of axonal mitochondria. Since eIF2β is conserved across many species, including *Drosophila* and humans, our results suggest that eIF2β may be a possible therapeutic target for aging and diseases associated with mitochondrial mislocalization.

## Materials and methods

### Fly stocks and husbandry

Flies were maintained in standard cornmeal medium (10% glucose, 0.7% agar, 9% cornmeal, 4% yeast extract, 0.3% propionic acid and 0.1% n-butyl p-hydroxybenzoate) at 25°C under light–dark cycles of 12:12 h. The flies were transferred to fresh food vials for every 2–3 days. UAS-milton RNAi (v41508) was from VDRC and outcrossed to [w1118] for five generations in our laboratory. Transgenic fly lines carrying UAS-*luciferase* RNAi control for *milton* RNAi) was reported previously^24^. GMR-gal4, Elav-gal4, UAS-*Pfk* RNAi (Bloomington stock center #36782), UAS-*luciferase* RNAi (Bloomington stock center #31603) (control for *Pfk* RNAi), UAS-*GFP* (used for control for UAS-*eIF2β*) and UAS-*eIF2β* (eIF2β^EY08063^, Bloomington stock center #17425) were from the Bloomington stock center. *eIF2β* heterozygous strain (PBac{SAstopDsRed} LL07719, DGRC#142114) was from KYOTO *Drosophila* Stock Center. UAS-*mitoGFP* was a kind gift from Drs. W. M. Saxton (University of California, Santa Cruz).

### Immunohistochemistry and image acquisition

Fly brains were dissected in PBS and fixed for 45 minutes in formaldehyde (4% v/v in PBS) at room temperature. After incubation in PBST containing 0.1% Triton X-100 for 10 min for three times, samples were incubated for 1h at room temperature in PBST containing 1% normal goat serum (Wako, Cat# 143-06561) and then incubated overnight with the primary antibody (anti-ubiquitin antibody Ubi-1 (1:50), anti-eIF2α (1:50) and anti-p-eIF2α (1:50)) diluted in 1% NGS/PBST at 4 °C. Samples were then washed for 10 min with PBST including 0.1% Triton X-100 three times and incubated with the secondary antibody for overnight at 4° C. Brains were mounted in Vectashield (Vectorlab Cat#H-1100) and analyzed under a confocal microscope (Nikon).

### Electron microscopy

Proboscis was removed from decapitated heads, which were then incubated in primary fixative solution (2.5% glutaraldehyde and 2% paraformaldehyde in 0.1 M sodium cacodylate buffer) at R.T. for 2 hours. After washing heads with 3% sucrose in 0.1 M sodium cacodylate buffer, fly heads were post-fixed for 1 hour in secondary fixation (1% osmium tetroxide in 0.1 M sodium cacodylate buffer) on ice. After washing with H_2_O, heads were dehydrated with ethanol and infiltrated with propylene oxide and Epon mixture (TAAB and Nissin EM) for 3 hours. After infiltration, specimens were embedded with Epon mixture at 70°C for 2~3 days. Thin-sections (70 nm) of laminas were collected on copper grids. The sections were stained with 5% uranyl acetate in 50% ethanol and Reynolds’ lead citrate solution. Electron micrographs were obtained with a CCD Camera mounted) on a JEM-1400 plus electron microscope (Jeol Ltd.).

### SDS–PAGE and immunoblotting

Western blotting was performed as reported previously^24^. Briefly, heads of 10-20 *Drosophila* were homogenized with SDS-Tris-Glycine sample buffer (0.312M Tris, 5% SDS, 8% glycerol, 0.0625% BPB, 10% β-mercaptoethanol, 10μ/mL leupeptin, 0.4 μM Pefabloc, 10mM β-glycerphosphate, 10mM NaF) and after boiling at 95°C for 2 minutes, it was centrifuged at 13,200 rpm, and the supernatant was used as a sample. To analyze p62 levels, fly heads were homogenized with 1% PBST and after centrifugation at 13,200 rpm, the supernatant was mixed 1:1 SDS-Tris-Glycine sample buffer, and boiled at 95°C for 2 minutes. The pellet was dissolved with 2% SDS in PBS, then centrifuged again at 13,200 rpm. The supernatant was mixed 1:1 SDS-Tris-Glycine sample buffer and then boiled at 95°C for 2 minutes. SDS–PAGE for western blotting was performed using 15%(w/v) (LC3), 7.5%(w/v) (p62), 10% (w/v) (eIF2α, β, and p-eIF2α) polyacrylamide gels. After electrophoresis, they were transferred to PVDF membrane (Merck Millipore) using a transfer device (BIO-RAD). After transfer, the membrane was blocked with 5% skim milk/TBST (50 mM Tris (pH 7.5), 0.15 M NaCl, 0.05% Tween20) for 1 hour and incubated with primary antibody listed below overnight at 4° C. Membranes were rinsed twice with TBST containing 0.65M NaCl and once with TBST containing 0.15M NaCl. After incubation with the secondary antibody at room temperature for 1 hour, membranes were rinsed twice with TBST containing 0.65M NaCl and once with TBST containing 0.15M NaCl. After incubation with Immobilon Western Chemiluminescent HRP Substrate (Merck Millipore), chemiluminescent signals were detected with Fusion FX (Vilber). Experiments were repeated at least 3 times with independent cohorts of flies. Primary antibodies: anti-LC3 antibody Atg8 (Merck Millipore #ABC975) (1:1000), anti-p62 antibody Ref2P (abcam #ab178440) (1:750), anti-eIF2β antibody (1:1500), anti-eIF2α antibody (abcam #ab26197) (1:1000), anti-p-eIF2α antibody (Cell signaling #3398S) (1:2000), anti-actin antibody (SIGMA A2066) (1:3000) and anti-β tubulin antibody (Sigma #T9026) (1:800,000). Polyclonal anti-eIF2β antibody was raised against a synthetic peptide (CGLEDDTKKEDPQDEA) corresponding to the C-terminal residues 29–43 of *Drosophila* eIF2β (1:1500).

Secondary antibodies: Peroxidase-conjugated goat anti-mouse IgG antibody (Dako #P0447) (1:2000), peroxidase-conjugated pig anti-rabbit IgG antibody (Dako #P0399) (1:2000)

### Proteasome assay

Heads from the 10 flies were homogenized in 150 μl of buffer B (25 mM Tris-HCl [pH 7.5], 2 mM ATP, 5 mM MgCl2, and 1 mM dithiothreitol). Proteasome peptidase activity in the lysates was measured with a synthetic peptide substrate, succinyl-Leu-Leu-Val-Tyr-7-amino-4-methyl-coumarin (Suc-LLVY-AMC) (Cayman). Luminescence was measured on an multimode plate reader 2300 Enspire (PerkinElmer). Experiments were repeated at least 3 times with independent cohorts of flies.

### ATP assay

Heads from the 10 flies were homogenized in 50 μl of 6 M guanidine-HCl in extraction buffer (100 mM Tris and 4 mM EDTA, pH 7.8) to inhibit ATPases. Samples were boiled for 5 min and centrifuged. Supernatant was diluted 4% with extraction buffer and mixed with a reaction solution (ATP Determination kit, Invitrogen). Luminescence was measured on a multimode plate reader 2300 Enspire (PerkinElmer). The relative ATP levels were calculated by dividing the luminescence by the total protein concentration, which was determined by the Bradford method. Experiments were repeated at least 3 times with independent cohorts of flies.

### Proteomic assay and pathway analysis

#### Sample preparation

Heads from the 35 flies were homogenized in 110 μl of extraction buffer (0.25% RapiGest SF, 50mM ammonium bicarbonate, 10mM dithiothreitol, 10μ/mL leupeptin, 0.4 μM Pefabloc, 10mM β-glycerphosphate, 10mM NaF). Homogenized samples were centrifugation and boiled for 5 min. After quantification of the protein concentration using a Pierce^®^ 660 nm Protein Assay (Thermo Fisher Scientific), 10 μg proteins from each sample were reduced using 5 mM tris (2-carboxyethyl) phosphine hydrochloride (TCEP-HCl; Thermo Fisher Scientific) at 60°C for 1 h, alkylated using 15 mM iodoacetamide (Fujifilm Wako Pure Chemical, Osaka, Japan) at room temperature for 30 min, and then digested using 1.5 μg Trypsin Gold (Mass Spectrometry Grade; Promega, Madison, WI, USA) at 37°C for 17 h. The digests were acidified by the addition of trifluoroacetic acid (TFA), incubated at 37°C for 30 min, and then centrifuged at 17,000 ×*g* for 10 min to remove the RapiGest SF. The supernatants were collected and desalted using MonoSpin^™^ C18 (GL Sciences, Tokyo, Japan). The resulting eluates were concentrated *in vacuo*, dissolved in 2% MeCN containing 0.1% formic acid (FA), and subjected to LC-MS/MS analysis.

#### LC-MS/MS analysis and database search

LC-MS/MS analyses were performed on an Ultimate 3000 RSLCnano system (Thermo Fisher Scientific) coupled to a Q Exactive hybrid quadrupole-Orbitrap mass spectrometer (Thermo Fisher Scientific) equipped with a nano electron spray ionization (ESI) source. The LC system was equipped with a trap column (C18 PepMap 100, 0.3 × 5 mm, 5 μm, Thermo Fisher Scientific) and an analytical column (NTCC-360/75-3-125, Nikkyo Technos, Tokyo, Japan). Peptide separation was performed using a 90-min gradient of water/0.1% FA (mobile phase A) and MeCN/0.1% FA (mobile phase B) at a flow rate of 300 nL/min. Elution was performed as follows: 0–3 min, 2% B; 3–93 min, 2%–40% B; 93–95 min, 40%–95% B; 95–105 min, 95% B; 105–107 min, 95%–2% B; and 107–120 min, 2% B. The mass spectrometer was operated in data-dependent acquisition mode. The MS parameters were as follows: spray voltage, 2.0 kV; capillary temperature, 275°C; S-lens RF level, 50; scan type, full MS; scan range, *m/z* 350–1500; resolution, 70,000; polarity, positive; automatic gain control target, 3 × 10^6^; and maximum injection time, 100 msec. The MS/MS parameters were as follows: resolution, 17,500; automatic gain control target, 1 × 10^5^; maximum injection time, 60 msec; normalized collision energy (NCE), 27; dynamic exclusion, 15 sec; loop count, 10; isolation window, 1.6 *m/z*; charge exclusion: unassigned, 1 and ≥8; and injection volume, 1 μL (containing 0.5 μg protein). Measurements were made in duplicate for each sample.

The identification of proteins and label-free quantification (LFQ) of the detected peptides were performed using Proteome Discoverer software ver. 2.4 (Thermo Fisher Scientific). The analytical parameters used for the database search were as follows: parent mass error tolerance, 10.0 ppm; fragment mass error tolerance, 0.02 Da; search engine, sequest HT; protein database, Drosophila melanogaster (Fruit fly: SwissProt Tax ID=7227); enzyme name, trypsin (full); maximum number of missed cleavages, 2; dynamic modification, oxidation (methionine), phosphorylation (serine, threonine, tyrosine), acetyl (lysine), GG (lysine); N-terminal modification, Met-loss (methionine), and Met-loss+acetyl (methionine); static modification, carbamidomethylation (cysteine) and FDR confidence, High < 0.01, 0.01 ≤ Medium < 0.05, 0.05 ≤ Low. The parameters for LFQ were as follows: precursor abundance, based on area; and normalization mode, total peptide amount. The abundance ratio of *milton* RNAi to control RNAi at 7 or 21 day-old was calculated. We considered proteins with the abundance ratio of ≥2.0 or ≤ 0.5 and an ANOVA *P*-value of < 0.05 based on volcano plots to be differentially expressed of *milton* RNAi. To extract molecular networks biologically relevant to the proteins differentially expressed due to *milton* RNAi, pathway analysis was performed using KeyMolnet (KM Data Inc., Tokyo, Japan).

### RNA extraction and quantitative real-time PCR analysis

Heads from more than 25 flies were mechanically isolated, and total RNA was extracted using ISOGEN (NipponGene) followed by reverse-transcription using PrimeScript RT reagent kit (Takara). The resulting cDNA was used as a template for PCR with THUNDERBIRD SYBR qPCR mix (TOYOBO) on a Thermal Cycler Dice real-time system TP800 (Takara). Expression of genes of interest was standardized relative to rp49. Relative expression values were determined by the ΔΔCT method (Livak and Schmittgen, 2001). Experiments were repeated three times, and a representative result was shown. Primers were designed using DRSC FlyPrimerBank (Harvard Medical School). Primer sequences are shown below;

*eIF2β* for 5′-GGACGACGACAAGAGCGAAG -3′

*eIF2β* rev 5′-CGGTCGCATCACGAACTTTG -3′

*milton* for 5′-GGCTTCAGGGCCAGGTATCT-3′

*milton* rev 5′-GCCGAACTTGGCTGACTTTG-3′

### Polysome gradient centrifugation

30 heads were homogenized in 150 μl of lysis buffer (25 mM Tris pH 7.5, 50 mM MgCl2, 250 mM NaCl, 1mM DTT, 0.5 mg/ml cycloheximide, 0.1 mg/ml heparin). The lysates were centrifuged at 13,200 rpm at 4 °C for 5 minutes and the supernatant was collected. The samples containing 38μ of RNA was layered gently on top of a 10–25% w/w sucrose gradient (50 mM Tris pH 7.5, 50 mM MgCl2, 250 mM NaCl, 0.1 mg/ml heparin, 0.5 mg/ml cycloheximide in 5 ml polyallomer tube) and centrifuged at 37,000 rpm at 4°C for 150 minutes in a himac CP-NX ultracentrifuge using a P50AT rotor. Samples were fractionated from top to bottom, and absorbance at OD260 nm was analyzed by a Plate reader (EnSpire). Experiments were repeated at least 3 times with independent cohorts of flies.

### Puromycin analysis

13 day-old flies were starved for 6 hr and fed 600 μM puromycin (Sigma) or 600 μM puromycin/35 mM cycloheximide (Sigma) in 5% sucrose solution for 20 hr. Incorporated puromycin was quantified by western blot with anti-puromycin antibody and normalized with actin. Experiments were repeated at least 3 times with independent cohorts of flies.

### Climbing assay

The climbing assay was performed as previously described^24^. Flies were placed in an empty plastic vial (2.5 cm in diameter × 10 cm in length). The vial was gently tapped to knock the flies to the bottom, and the number of flies reached to the top, middle and bottom areas of the vials in 10 s was counted. Experiments were repeated 10 times, and the mean percentage of flies in each area and standard deviations was calculated. Experiments were repeated with independent cohorts more than three times, and a representative result was shown.

### Statistics

The number of replicates, what n represents, precision measurements, and the meaning of error bars are indicated in Figure Legends. Data are shown as means ± SEM. For pairwise comparisons, Student’s t-test was performed with Microsoft Excel (Microsoft). For multiple comparisons, data were analyzed using one-way ANOVA with Tukey’s HSD multiple-comparisons test in the GraphPad Prism 6.0 software (GraphPad Software, Inc., La Jolla, CA). Results with a p-value of less than 0.05 were considered to be statistically significant.

## Supplementary Material

1

## Figures and Tables

**Table 1 :** Differentially expressed proteins in *milton* RNAi fly brains compared to control at 7 day-old detected by proteome analysis

7 day-old			
Accession^a^	Name	Abundance Ratio	Abundance Ratio P-Value
Q9V751	Attacin-B	100	1E-17
Q04448	Bifunctional methylenetetrahydrofolate dehydrogenase/cyclohydrolase, mitochondrial	100	1E-17
P22700	Calcium-transporting ATPase sarcoplasmic/endoplasmic reticulum type	100	1E-17
Q9V558	Cytochrome P450 4p1	100	1E-17
P10552	FMRFamide-related peptides	100	1E-17
P05661-19	Isoform F of Myosin heavy chain, muscle	100	1E-17
Q9VE01	Probable cytochrome P450 12a5, mitochondrial	100	1E-17
Q7KIN0	Toll-like receptor 7	100	1E-17
Q8MKN0	Ubiquinone biosynthesis protein COQ9, mitochondrial	100	1E-17
Q9VJG0	Xaa-Pro aminopeptidase ApepP	100	1E-17
Q9V8F5	Bomanin Bicipital 1	4.908	1E-17
P07701	Salivary glue protein Sgs-5	2.843	1.9925E-09
O76902	Pleckstrin homology domain-containing family F member 1 homolog	2.836	2.2204E-16
P81641	Alpha-amylase B	2.684	7.4485E-09
P19351-4	Isoform 4 of Troponin T, skeletal muscle	2.66	6.6556E-11
Q9VTJ8	Mitochondrial import inner membrane translocase subunit TIM14	2.61	3.5421E-07
P41375	Eukaryotic translation initiation factor 2 subunit 2	2.465	3.3849E-09
Q9VYB0	Selenoprotein BthD	2.462	2.2558E-08
B7Z0W9	Proton channel OtopLc	2.382	1.7174E-06
Q9VLR5	RNA polymerase II transcriptional coactivator	2.245	6.7024E-09
Q8IN44	Protein Turandot A	2.127	8.3866E-13
P27779	Pupal cuticle protein Edg-78E	2.113	1.0021E-08
Q9W1X8	Probable GDP-L-fucose synthase	0.496	2.126E-08
P55035	26S proteasome non-ATPase regulatory subunit 4	0.487	5.5016E-11
Q9VHN6	39S ribosomal protein L19, mitochondrial	0.487	0.00055134
Q9VPD2	Cytosolic Fe-S cluster assembly factor NUBP2 homolog	0.46	6.3451E-05
Q9VHD3	Probable maleylacetoacetate isomerase 1	0.432	4.5096E-06
Q94529	Probable pseudouridine-5’-phosphatase	0.416	1.088E-14
Q27606	Cytochrome P450 4e2	0.398	4.1313E-10
Q24388	Larval serum protein 2	0.378	1.3323E-14
Q9VKH6	Lysosomal thioesterase PPT2 homolog	0.369	2.0523E-06
Q24114	Division abnormally delayed protein	0.307	2.2441E-09
Q95NH6	Attacin-C	0.01	1E-17
P29993	Inositol 1,4,5-trisphosphate receptor	0.01	1E-17
Q94526	Open rectifier potassium channel protein 1	0.01	1E-17
Q9Y115	UNC93-like protein	0.01	1E-17
21 day-old			
Accession^a^	Name	Abundance Ratio	Abundance Ratio P-Value
Q10714	Angiotensin-converting enzyme	100	1E-17
C0HKQ8	Cecropin-A2	100	1E-17
Q9V558	Cytochrome P450 4p1	100	1E-17
P51592	E3 ubiquitin-protein ligase hyd	100	1E-17
Q9VMJ7	Lysine-specific demethylase lid	100	1E-17
Q9VXP4	Platelet-activating factor acetylhydrolase IB subunit beta homolog	100	1E-17
Q9VY28	Probable 28S ribosomal protein S25, mitochondrial	100	1E-17
Q9W391	Probable phosphorylase b kinase regulatory subunit alpha	100	1E-17
Q9VUQ5	Protein argonaute-2	100	1E-17
P54359	Septin-2	100	1E-17
P24492	Diptericin A	15.716	1E-17
Q9VVY3	Glycogen-binding subunit 76A	8.986	1E-17
Q70PY2	Peptidoglycan-recognition protein SB1	6.669	1E-17
Q9W0M1	Centrosomal protein cep290	6.526	1E-17
P81641	Alpha-amylase B	5.722	1E-17
P45884	Attacin-A	4.997	1E-17
C0HL66	Histone H3.3A	4.778	1E-17
P26675	Protein son of sevenless	4.696	1E-17
P02515	Heat shock protein 22	4.69	1E-17
Q95NH6	Attacin-C	4.35	1E-17
P17971-1	Isoform A of Potassium voltage-gated channel protein Shal	4.195	1E-17
Q7K1U0	Activity-regulated cytoskeleton associated protein 1	3.271	1E-17
P14199	Protein ref(2)P	3.014	1E-17
Q9VU02	Probable small nuclear ribonucleoprotein Sm D1	2.43	4.91607E-13
Q9VD44	Poly(A) RNA polymerase gld-2 homolog A	2.268	2.6084E-11
Q9V8F5	Bomanin Bicipital 1	2.24	5.16671E-11
P22979	Heat shock protein 67B3	2.223	7.90048E-11
P27779	Pupal cuticle protein Edg-78E	2.192	1.65944E-10
Q9NBK5	Serine/threonine-protein kinase tricornered	2.059	4.15071E-09
Q8MLZ7	Chitinase-like protein Idgf3	2.055	4.61182E-09
Q9V751	Attacin-B	2.038	6.79416E-09
Q9V8M5	Probable 3-hydroxyisobutyrate dehydrogenase, mitochondrial	0.492	7.42952E-09
P84345	ATP synthase protein 8	0.421	1.809E-12
P33438	Glutactin	0.414	6.13731E-13
Q8IN44	Protein Turandot A	0.218	1E-17
Q8IN43	Protein Turandot C	0.195	1E-17
Q9VFI9	cGMP-specific 3’,5’-cyclic phosphodiesterase	0.01	1E-17
Q94526	Open rectifier potassium channel protein 1	0.01	1E-17
Q9VHD3	Probable maleylacetoacetate isomerase 1	0.01	1E-17
Q9W0A0	Protein draper	0.01	1E-17
A1Z7T0	Serine/threonine-protein kinase N	0.01	1E-17

a UniProt accession number

**Table 2 :** Molecule networks based on “Interaction search” of KeyMolnet

7 day-old					
Rank	Name	Score	Score (p) ^a^	Score (v) ^b^	Score (c) ^c^
1	Autophagy-related protein signaling pathway	50.394	6.76E-16	0.159	0.11
2	Calcium signaling pathway	47.583	4.75E-15	0.146	0.117
3	Transcriptional regulation by SMAD	44.012	5.64E-14	0.146	0.095
4	GABA signaling pathway	40.706	5.58E-13	0.122	0.123
5	estrogen signaling pathway	37.507	5.12E-12	0.11	0.13
6	Sirtuin signaling pathway	36.87	7.96E-12	0.122	0.095
7	Transcriptional regulation by AP-1	34.874	3.18E-11	0.11	0.107
8	Arrestin signaling pathway	32.84	1.30E-10	0.11	0.092
9	G protein (Gq/11) signaling pathway	30.889	5.03E-10	0.085	0.149
10	Kainate receptor signaling pathway	30.049	9.00E-10	0.073	0.214
11	Transcriptional regulation by C/EBP	29.5	1.32E-09	0.098	0.093
12	Calpain signaling pathway	28.597	2.46E-09	0.11	0.066
13	Phospholipase D signaling pathway	28.344	2.94E-09	0.098	0.084
14	HSP90 signaling pathwa^y^	27.188	6.54E-09	0.085	0.104
14	CYP family	27.188	6.54E-09	0.085	0.104
16	Kir3 channel signaling pathwa	26.495	1.06E-08	0.061	0.25
17	Estrogen biosynthesis	26.107	1.38E-08	0.061	0.238
18	CaSR signaling pathway	25.39	2.27E-08	0.061	0.217
19	PI3K signaling pathway	24.927	3.14E-08	0.073	0.122
20	PAF receptor signaling pathway	24.555	4.06E-08	0.049	0.4
21	Transcriptional regulation by PPARa	24.398	4.52E-08	0.073	0.115
21	BTK signaling pathway	24.398	4.52E-08	0.073	0.115
23	Transcriptional regulation by STAT	24.076	5.66E-08	0.085	0.077
24	G protein (Gi/o) signaling pathway	24.063	5.71E-08	0.073	0.111
25	PARP signaling pathway	23.742	7.13E-08	0.073	0.107
25	mGluR signaling pathway	23.742	7.13E-08	0.073	0.107
27	Free fatty acid signaling pathway	23.433	8.83E-08	0.073	0.103
28	Kir channel signaling pathway	23.338	9.43E-08	0.061	0.167
29	Oxytocin signaling pathway	23.327	9.51E-08	0.049	0.333
30	Transcriptional regulation by MEF2	22.99	1.20E-07	0.073	0.098
31	S100 family signaling pathway	22.434	1.77E-07	0.073	0.092
32	Transcriptional regulation by FOXO	22.301	1.94E-07	0.073	0.091
33	P2Y signaling pathway	22.172	2.12E-07	0.061	0.143
34	Transcriptional regulation by SRF	21.174	4.23E-07	0.061	0.125
34	ATF4/ATF6/IRE1 signaling pathway	21.174	4.23E-07	0.061	0.125
36	Chemerin signaling pathway	21.082	4.50E-07	0.049	0.235
36	Vasopressin signaling pathway	21.082	4.50E-07	0.049	0.235
38	Serotonin signaling pathway	20.854	5.28E-07	0.073	0.077
39	Transcriptional regulation by HIF	20.834	5.35E-07	0.098	0.043
40	Leukotriene receptorsignaling pathway	20.724	5.78E-07	0.049	0.222
40	CART signaling pathway	20.724	5.78E-07	0.049	0.222
42	MAPK signaling pathway	20.693	5.90E-07	0.085	0.055
43	Transcriptional regulation by RB/E2F	20.543	6.55E-07	0.098	0.042
44	NAD metabolism	20.468	6.89E-07	0.061	0.114
45	ERK signaling pathway	20.425	7.11E-07	0.073	0.073
46	Adenylyl Cyclase signaling pathway	20.303	7.73E-07	0.061	0.111
47	Bile acid signaling pathway	20.141	8.65E-07	0.061	0.109
21 day-old					
Rank	Name	Score	Score (p) ^a^	Score (v) ^b^	Score (c) ^c^
1	Histone demethylation	84.198	4.51E-26	0.102	0.425
2	CDK inhibitor signaling pathway	56.497	9.83E-18	0.078	0.295
3	Transcriptional regulation by RB/E2F	46.598	9.39E-15	0.108	0.095
4	Mst(Hippo) signaling pathway)	46.343	1.12E-14	0.09	0.133
5	Transcriptional regulation by androgen receptor	46.078	1.35E-14	0.078	0.178
6	p160 SRC signaling pathway	45.809	1.62E-14	0.078	0.176
7	Transcriptional regulation by SMAD	43.961	5.84E-14	0.09	0.119
8	Autophagy-related protein signaling pathway	41.063	4.35E-13	0.084	0.119
9	Transcriptional regulation by HIF	39.527	1.26E-12	0.096	0.086
10	Nucleophosmin signaling pathway	38.417	2.72E-12	0.054	0.273
11	HSP90 signaling pathway	37.887	3.93E-12	0.066	0.164
12	PAF metabolism	37.562	4.93E-12	0.042	0.5
13	Transcriptional regulation by STAT	37.276	6.01E-12	0.072	0.132
14	BcI-2 family signaling pathway	36.157	1.31E-11	0.072	0.124
15	Sirtuin signaling pathway	34.782	3.39E-11	0.072	0.114
16	Transcriptional regulation by C/EBP	33.819	6.60E-11	0.066	0.128
17	PIN1 signaling pathway	33.172	1.03E-10	0.06	0.149
18	RSK signaling pathway	30.566	6.29E-10	0.06	0.125
19	Transcriptional regulation by High mobility group protein	29.873	1.02E-09	0.054	0.148
20	BET family signaling pathway	29.656	1.18E-09	0.054	0.145
21	Transcriptional regulation by Myc	28.838	2.08E-09	0.066	0.093
22	Transcriptional regulation by FOXO	28.827	2.10E-09	0.054	0.136
23	PSD-95 family signaling pathway	26.154	1.34E-08	0.048	0.14
24	AKT signaling pathway	25.169	2.65E-08	0.048	0.129
25	Arginine methylation	24.799	3.43E-08	0.048	0.125
26	gp130 signaling pathway	24.25	5.01E-08	0.054	0.096
27	Transcriptional regulation by CREB	23.858	6.58E-08	0.066	0.067
28	Gene regulation by microRNAs (metastasis)	23.852	6.60E-08	0.054	0.093
29	HDAC signaling pathway	23.536	8.22E-08	0.036	0.207
30	Calpain signaling pathway	23.092	1.12E-07	0.06	0.074
31	Transcriptional regulation by IRF	22.738	1.43E-07	0.054	0.085
32	2-Oxoglutarate signaling pathway	22.673	1.50E-07	0.048	0.104
32	14-3-3 signaling pathway	22.673	1.50E-07	0.048	0.104
34	Transcriptional regulation by POU domain factor	22.601	1.57E-07	0.06	0.071
35	Transcriptional regulation by BLIMP-1	22.474	1.72E-07	0.042	0.132
36	Gene regulation by microRNAs (metabolism)	22.39	1.82E-07	0.054	0.083
37	Fatty acid beta oxidation	22.096	2.23E-07	0.042	0.127
38	Transcriptional regulation by RXR	22.08	2.26E-07	0.036	0.176
39	ERK signaling pathway	21.96	2.45E-07	0.048	0.098
40	PARP signaling pathway	21.913	2.53E-07	0.042	0.125
41	Transcriptional regulation by VDR	21.618	3.11E-07	0.054	0.078
42	Transcriptional regulation by p53	21.168	4.24E-07	0.072	0.05
43	Acetylcholine metabolism	21.152	4.29E-07	0.024	0.444
44	Gene regulation by microRNAs (embryonic stem cells)	21.08	4.51E-07	0.036	0.158
45	mTOR signaling pathway	21.048	4.61E-07	0.042	0.115
46	Gene regulation by microRNAs (cancer)	21.04	4.64E-07	0.048	0.09
47	Transcriptional regulation by Ets-1/2	20.724	5.77E-07	0.042	0.111
48	MAPK signaling pathway	20.411	7.18E-07	0.054	0.07
49	Gene regulation by microRNAs (cell cycle)	20.404	7.21E-07	0.036	0.146
50	Transcriptional regulation by p73	20.259	7.97E-07	0.042	0.106

a Score(p) indicates p-value of the pathway.

b Score(v) indicates the ratio of “Count” to total molecules associated with the loaded list.

c Score(c) indicates the ratio of “Count” to total molecules contained in the pathway.

## Data Availability

The datasets used and/or analyzed during the current study are available from the corresponding author upon request.

## Highlights

- Loss of axonal mitochondria impairs autophagy and accumulates proteins in the axon
- Loss of axonal mitochondria upregulates eIF2β and downregulates p-eIF2α
- Neuronal upregulation of eIF2β induces autophagic defects and locomotor dysfunction
- Lowering eIF2β rescues autophagic defects caused by loss of axonal mitochondria
