# Supplementary figures and images for: Axonal distribution of mitochondria maintains neuronal autophagy during aging via eIF2β
Source: bioRxiv. 2025 Oct 1:2024.01.20.576435. Originally published 2024 Jan 20. Preprint. [Version 4] doi: 10.1101/2024.01.20.576435 (PMC10827206; doi:10.1101/2024.01.20.576435)

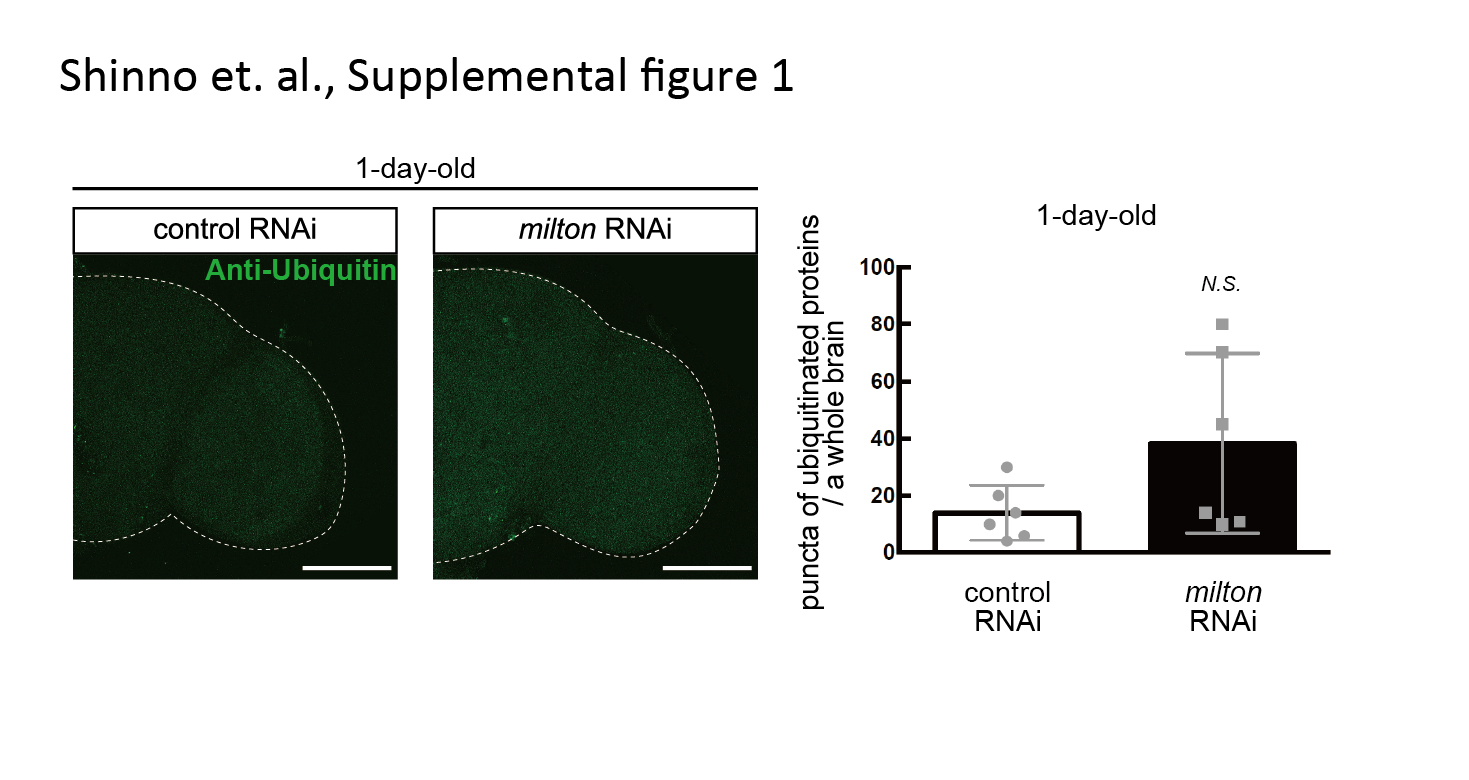

Supplement: Supplement 2 — Supplemental Figure 1. Ubiquitinated proteins in brains with neuronal knockdown of milton at 1-day-old Brains dissected at 1-day-old were immunostained with an antibody against ubiquitin. Representative images (left) and quantitation of the number of ubiquitin-positive puncta (right) are shown. Scale bars of hemibrains, 100 μm, Means ± SE, n = 8. N.S., p > 0.05 (Student’s t-test). [file media-2.tif]

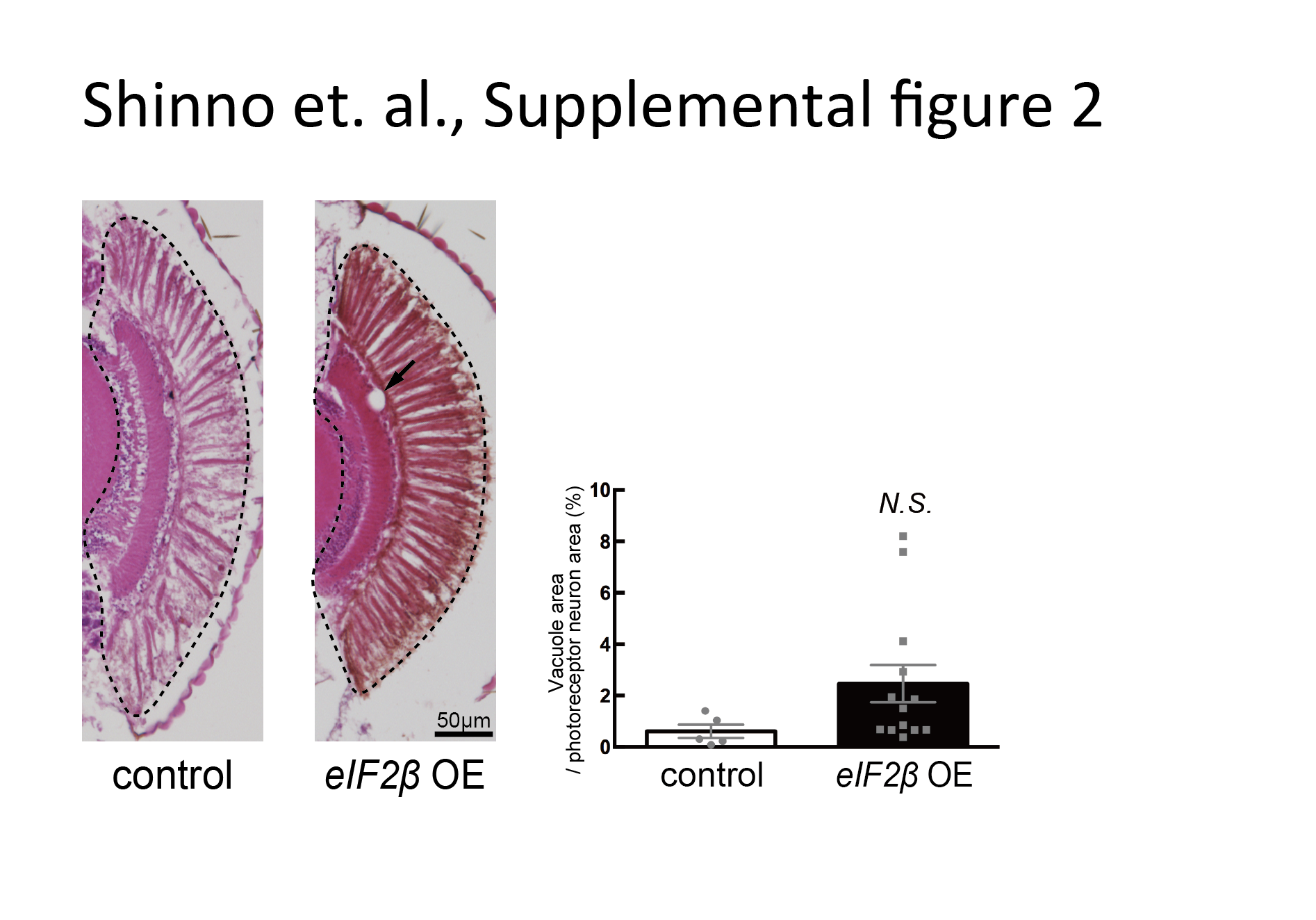

Supplement: Supplement 3 — Supplemental Figure 2. Histology analysis of fly heads with eIF2β overexpression The morphology of the eye with eIF2β overexpression. The dotted lines indicate the retina. Vacuoles are indicated by arrows. Representative images (left) and quantification of vacuole area (right). The flies were 40-day-old. Means ± SE, n = 5–13 N.S., p > 0.05 (Student’s t-test). [file media-3.tif]
