# Supplementary material for: Axonal distribution of mitochondria maintains neuronal autophagy during aging via eIF2β
Source: bioRxiv. 2024 Dec 31:2024.01.20.576435. Originally published 2024 Jan 20. Preprint. [Version 2] doi: 10.1101/2024.01.20.576435 (PMC10827206; doi:10.1101/2024.01.20.576435)
Supplement: Supplementary file 1 [file NIHPP2024.01.20.576435V2-supplement-1.pdf]

bioRxiv preprint doi: https://doi.org/10.1101/2024.01.20.576435; this version posted December 31, 2024. The copyright holder for this preprint (which was not certified by peer review) is the author/funder, who has granted bioRxiv a license to display the preprint in perpetuity. It is made available under aCC-BY 4.0 International license.

7 day-old			
Accession[a]	Name	Abundance Ratio: (7 days, milton KD) / (7 days, control)	Abundance Ratio P-Value: (7 days, milton KD) / (7 days, control)
Q9V751	Attacin-B	100	1E-17
Q04448	Bifunctional methylenetetrahydrofolate dehydrogenase/cyclohydrolase, mitochondrial	100	1E-17
P22700	Calcium-transporting ATPase sarcoplasmic/endoplasmic reticulum type	100	1E-17
Q9V558	Cytochrome P450 4p1	100	1E-17
P10552	FMRFamide-related peptides	100	1E-17
P05661-19	Isoform F of Myosin heavy chain, muscle	100	1E-17
Q9VE01	Probable cytochrome P450 12a5, mitochondrial	100	1E-17
Q7KIN0	Toll-like receptor 7	100	1E-17
Q8MKN0	Ubiquinone biosynthesis protein COQ9, mitochondrial	100	1E-17
Q9VJG0	Xaa-Pro aminopeptidase ApepP	100	1E-17
Q9V8F5	Bomanin Bicipital 1	4.908	1E-17
P07701	Salivary glue protein Sgs-5	2.843	1.99252E-09
O76902	Pleckstrin homology domain-containing family F member 1 homolog	2.836	2.22045E-16
P81641	Alpha-amylase B	2.684	7.44847E-09
P19351-4	Isoform 4 of Troponin T, skeletal muscle	2.66	6.65563E-11
Q9VTJ8	Mitochondrial import inner membrane translocase subunit TIM14	2.61	3.54205E-07
P41375	Eukaryotic translation initiation factor 2 subunit 2	2.465	3.38486E-09
Q9VYB0	Selenoprotein BthD	2.462	2.25579E-08
B7Z0W9	Proton channel OtopLc	2.382	1.71741E-06
Q9VLR5	RNA polymerase II transcriptional coactivator	2.245	6.70245E-09
Q8IN44	Protein Turandot A	2.127	8.38662E-13
P27779	Pupal cuticle protein Edg-78E	2.113	1.00215E-08
Q9W1X8	Probable GDP-L-fucose synthase	0.496	2.12601E-08
P55035	26S proteasome non-ATPase regulatory subunit 4	0.487	5.50158E-11
Q9VHN6	39S ribosomal protein L19, mitochondrial	0.487	0.000551337
Q9VPD2	Cytosolic Fe-S cluster assembly factor NUBP2 homolog	0.46	6.34514E-05
Q9VHD3	Probable maleylacetoacetate isomerase 1	0.432	4.50956E-06
Q94529	Probable pseudouridine-5'-phosphatase	0.416	1.08802E-14
Q27606	Cytochrome P450 4e2	0.398	4.13128E-10
Q24388	Larval serum protein 2	0.378	1.33227E-14
Q9VKH6	Lysosomal thioesterase PPT2 homolog	0.369	2.05225E-06
Q24114	Division abnormally delayed protein	0.307	2.24406E-09
Q95NH6	Attacin-C	0.01	1E-17
P29993	Inositol 1,4,5-trisphosphate receptor	0.01	1E-17
Q94526	Open rectifier potassium channel protein 1	0.01	1E-17
Q9Y115	UNC93-like protein	0.01	1E-17

[a] UniProt accession number

21 day-old			
Accession[a]	Name	Abundance Ratio: (21 days, Milton KD) / (21 days, Control)	Abundance Ratio P-Value: (21 days, Milton KD) / (21 days, Control)
Q10714	Angiotensin-converting enzyme	100	1E-17
C0HKQ8	Cecropin-A2	100	1E-17
Q9V558	Cytochrome P450 4p1	100	1E-17
P51592	E3 ubiquitin-protein ligase hyd	100	1E-17
Q9VMJ7	Lysine-specific demethylase lid	100	1E-17
Q9VXP4	Platelet-activating factor acetylhydrolase IB subunit beta homolog	100	1E-17
Q9VY28	Probable 28S ribosomal protein S25, mitochondrial	100	1E-17
Q9W391	Probable phosphorylase b kinase regulatory subunit alpha	100	1E-17
Q9VUQ5	Protein argonaute-2	100	1E-17
P54359	Septin-2	100	1E-17
P24492	Diptericin A	15.716	1E-17
Q9VVY3	Glycogen-binding subunit 76A	8.986	1E-17
Q70PY2	Peptidoglycan-recognition protein SB1	6.669	1E-17
Q9W0M1	Centrosomal protein cep290	6.526	1E-17
P81641	Alpha-amylase B	5.722	1E-17
P45884	Attacin-A	4.997	1E-17
C0HL66	Histone H3.3A	4.778	1E-17
P26675	Protein son of sevenless	4.696	1E-17
P02515	Heat shock protein 22	4.69	1E-17
Q95NH6	Attacin-C	4.35	1E-17
P17971-1	Isoform A of Potassium voltage-gated channel protein Shal	4.195	1E-17
Q7K1U0	Activity-regulated cytoskeleton associated protein 1	3.271	1E-17
P14199	Protein ref(2)P	3.014	1E-17
Q9VU02	Probable small nuclear ribonucleoprotein Sm D1	2.43	4.91607E-13
Q9VD44	Poly(A) RNA polymerase gld-2 homolog A	2.268	2.6084E-11
Q9V8F5	Bomanin Bicipital 1	2.24	5.16671E-11
P22979	Heat shock protein 67B3	2.223	7.90048E-11
P27779	Pupal cuticle protein Edg-78E	2.192	1.65944E-10
Q9NBK5	Serine/threonine-protein kinase tricornered	2.059	4.15071E-09
Q8MLZ7	Chitinase-like protein Idgf3	2.055	4.61182E-09
Q9V751	Attacin-B	2.038	6.79416E-09
Q9V8M5	Probable 3-hydroxyisobutyrate dehydrogenase, mitochondrial	0.492	7.42952E-09
P84345	ATP synthase protein 8	0.421	1.809E-12
P33438	Glutactin	0.414	6.13731E-13
Q8IN44	Protein Turandot A	0.218	1E-17
Q8IN43	Protein Turandot C	0.195	1E-17
Q9VFI9	cGMP-specific 3',5'-cyclic phosphodiesterase	0.01	1E-17
Q94526	Open rectifier potassium channel protein 1	0.01	1E-17
Q9VHD3	Probable maleylacetoacetate isomerase 1	0.01	1E-17
Q9W0A0	Protein draper	0.01	1E-17
A1Z7T0	Serine/threonine-protein kinase N	0.01	1E-17

bioRxiv preprint doi: https://doi.org/10.1101/2024.01.20.576435; this version posted December 31, 2024. The copyright holder for this preprint (which was not certified by peer review) is the author/funder, who has granted bioRxiv a license to display the preprint in perpetuity. It is made available under aCC-BY 4.0 International license.

7 day-old					
Rank	Name	Score	Score (p) [a]	Score (v) [b]	Score (c) [c]
1	Autophagy-related protein signaling pathway	50.394	6.76E-16	0.159	0.11
2	Calcium signaling pathway	47.583	4.75E-15	0.146	0.117
3	Transcriptional regulation by SMAD	44.012	5.64E-14	0.146	0.095
4	GABA signaling pathway	40.706	5.58E-13	0.122	0.123
5	estrogen signaling pathway	37.507	5.12E-12	0.11	0.13
6	Sirtuin signaling pathway	36.87	7.96E-12	0.122	0.095
7	Transcriptional regulation by AP-1	34.874	3.18E-11	0.11	0.107
8	Arrestin signaling pathway	32.84	1.30E-10	0.11	0.092
9	G protein (Gq/11) signaling pathway	30.889	5.03E-10	0.085	0.149
10	Kainate receptor signaling pathway	30.049	9.00E-10	0.073	0.214
11	Transcriptional regulation by C/EBP	29.5	1.32E-09	0.098	0.093
12	Calpain signaling pathway	28.597	2.46E-09	0.11	0.066
13	Phospholipase D signaling pathway	28.344	2.94E-09	0.098	0.084
14	HSP90 signaling pathway	27.188	6.54E-09	0.085	0.104
14	CYP family	27.188	6.54E-09	0.085	0.104
16	Kir3 channel signaling pathway	26.495	1.06E-08	0.061	0.25
17	Estrogen biosynthesis	26.107	1.38E-08	0.061	0.238
18	CaSR signaling pathway	25.39	2.27E-08	0.061	0.217
19	PI3K signaling pathway	24.927	3.14E-08	0.073	0.122
20	PAF receptor signaling pathway	24.555	4.06E-08	0.049	0.4
21	Transcriptional regulation by PPARa	24.398	4.52E-08	0.073	0.115
21	BTK signaling pathway	24.398	4.52E-08	0.073	0.115
23	Transcriptional regulation by STAT	24.076	5.66E-08	0.085	0.077
24	G protein (Gi/o) signaling pathway	24.063	5.71E-08	0.073	0.111
25	PARP signaling pathway	23.742	7.13E-08	0.073	0.107
25	mGluR signaling pathway	23.742	7.13E-08	0.073	0.107
27	Free fatty acid signaling pathway	23.433	8.83E-08	0.073	0.103
28	Kir channel signaling pathway	23.338	9.43E-08	0.061	0.167
29	Oxytocin signaling pathway	23.327	9.51E-08	0.049	0.333
30	Transcriptional regulation by MEF2	22.99	1.20E-07	0.073	0.098
31	S100 family signaling pathway	22.434	1.77E-07	0.073	0.092
32	Transcriptional regulation by FOXO	22.301	1.94E-07	0.073	0.091
33	P2Y signaling pathway	22.172	2.12E-07	0.061	0.143
34	Transcriptional regulation by SRF	21.174	4.23E-07	0.061	0.125
34	ATF4/ATF6/IRE1 signaling pathway	21.174	4.23E-07	0.061	0.125
36	Chemerin signaling pathway	21.082	4.50E-07	0.049	0.235
36	Vasopressin signaling pathway	21.082	4.50E-07	0.049	0.235
38	Serotonin signaling pathway	20.854	5.28E-07	0.073	0.077
39	Transcriptional regulation by HIF	20.834	5.35E-07	0.098	0.043
40	Leukotriene receptorsignaling pathway	20.724	5.78E-07	0.049	0.222
40	CART signaling pathway	20.724	5.78E-07	0.049	0.222
42	MAPK signaling pathway	20.693	5.90E-07	0.085	0.055
43	Transcriptional regulation by RB/E2F	20.543	6.55E-07	0.098	0.042
44	NAD metabolism	20.468	6.89E-07	0.061	0.114
45	ERK signaling pathway	20.425	7.11E-07	0.073	0.073
46	Adenylyl Cyclase signaling pathway	20.303	7.73E-07	0.061	0.111
47	Bile acid signaling pathway	20.141	8.65E-07	0.061	0.109

[a] Score(p) indicates p-value of the pathway.
[b] Score(v) indicates the ratio of "Count" to total molecules associated with the loaded list.
[c] Score(c) indicates the ratio of "Count" to total molecules contained in the pathway.

21 day-old					
Rank	Name	Score	Score (p) [a]	Score (v) [b]	Score (c) [c]
1	Histone demethylation	84.198	4.51E-26	0.102	0.425
2	CDK inhibitor signaling pathway	56.497	9.83E-18	0.078	0.295
3	Transcriptional regulation by RB/E2F	46.598	9.39E-15	0.108	0.095
4	Mst(Hippo) signaling pathway)	46.343	1.12E-14	0.09	0.133
5	Transcriptional regulation by androgen receptor	46.078	1.35E-14	0.078	0.178
6	p160 SRC signaling pathway	45.809	1.62E-14	0.078	0.176
7	Transcriptional regulation by SMAD	43.961	5.84E-14	0.09	0.119
8	Autophagy-related protein signaling pathway	41.063	4.35E-13	0.084	0.119
9	Transcriptional regulation by HIF	39.527	1.26E-12	0.096	0.086
10	Nucleophosmin signaling pathway	38.417	2.72E-12	0.054	0.273
11	HSP90 signaling pathway	37.887	3.93E-12	0.066	0.164
12	PAF metabolism	37.562	4.93E-12	0.042	0.5
13	Transcriptional regulation by STAT	37.276	6.01E-12	0.072	0.132
14	Bcl-2 family signaling pathway	36.157	1.31E-11	0.072	0.124
15	Sirtuin signaling pathway	34.782	3.39E-11	0.072	0.114
16	Transcriptional regulation by C/EBP	33.819	6.60E-11	0.066	0.128
17	PIN1 signaling pathway	33.172	1.03E-10	0.06	0.149
18	RSK signaling pathway	30.566	6.29E-10	0.06	0.125
19	Transcriptional regulation by High mobility group protein	29.873	1.02E-09	0.054	0.148
20	BET family signaling pathway	29.656	1.18E-09	0.054	0.145
21	Transcriptional regulation by Myc	28.838	2.08E-09	0.066	0.093
22	Transcriptional regulation by FOXO	28.827	2.10E-09	0.054	0.136
23	PSD-95 family signaling pathway	26.154	1.34E-08	0.048	0.14
24	AKT signaling pathway	25.169	2.65E-08	0.048	0.129
25	Arginine methylation	24.799	3.43E-08	0.048	0.125
26	gp130 signaling pathway	24.25	5.01E-08	0.054	0.096
27	Transcriptional regulation by CREB	23.858	6.58E-08	0.066	0.067
28	Gene regulation by microRNAs (metastasis)	23.852	6.60E-08	0.054	0.093
29	HDAC signaling pathway	23.536	8.22E-08	0.036	0.207
30	Calpain signaling pathway	23.092	1.12E-07	0.06	0.074
31	Transcriptional regulation by IRF	22.738	1.43E-07	0.054	0.085
32	2-Oxoglutarate signaling pathway	22.673	1.50E-07	0.048	0.104
32	14-3-3 signaling pathway	22.673	1.50E-07	0.048	0.104
34	Transcriptional regulation by POU domain factor	22.601	1.57E-07	0.06	0.071
35	Transcriptional regulation by BLIMP-1	22.474	1.72E-07	0.042	0.132
36	Gene regulation by microRNAs (metabolism)	22.39	1.82E-07	0.054	0.083
37	Fatty acid beta oxidation	22.096	2.23E-07	0.042	0.127
38	Transcriptional regulation by RXR	22.08	2.26E-07	0.036	0.176
39	ERK signaling pathway	21.96	2.45E-07	0.048	0.098
40	PARP signaling pathway	21.913	2.53E-07	0.042	0.125
41	Transcriptional regulation by VDR	21.618	3.11E-07	0.054	0.078
42	Transcriptional regulation by p53	21.168	4.24E-07	0.072	0.05
43	Acetylcholine metabolism	21.152	4.29E-07	0.024	0.444
44	Gene regulation by microRNAs (embryonic stem cells)	21.08	4.51E-07	0.036	0.158
45	mTOR signaling pathway	21.048	4.61E-07	0.042	0.115
46	Gene regulation by microRNAs (cancer)	21.04	4.64E-07	0.048	0.09
47	Transcriptional regulation by Ets-1/2	20.724	5.77E-07	0.042	0.111
48	MAPK signaling pathway	20.411	7.18E-07	0.054	0.07
49	Gene regulation by microRNAs (cell cycle)	20.404	7.21E-07	0.036	0.146
50	Transcriptional regulation by p73	20.259	7.97E-07	0.042	0.106
