# Supplementary material for: Axonal distribution of mitochondria maintains neuronal autophagy during aging via eIF2β
Source: bioRxiv. 2024 Jan 20:2024.01.20.576435. Preprint. [Version 1] doi: 10.1101/2024.01.20.576435 (PMC10827206; doi:10.1101/2024.01.20.576435)
Supplement: Supplementary file 1 [file NIHPP2024.01.20.576435v1-supplement-1.pdf]

bioRxiv preprint doi: https://doi.org/10.1101/2024.01.20.576435; this version posted January 20, 2024. The copyright holder for this preprint (which was not certified by peer review) is the author/funder, who has granted bioRxiv a license to display the preprint in perpetuity. It is made available under aCC-BY 4.0 International license.

Supplemental Figure 1. Western blot analysis of eIF2α and p-eIF2α with *milton* knockdown

Western blotting of head extracts with anti-eIF2α (A) and anti-p-eIF2α (B) antibodies. Flies were 14 day-old. Representative blots (left) and quantitation (right) are shown.

bioRxiv preprint doi: https://doi.org/10.1101/2024.01.20.576435; this version posted January 20, 2024. The copyright holder for this preprint (which was not certified by peer review) is the author/funder, who has granted bioRxiv a license to display the preprint in perpetuity. It is made available under aCC-BY 4.0 International license.

Tubulin was used as a loading control. Means ± SE, n = 6. *N.S.*, $p > 0.05$; *$p < 0.05$ (Student's *t*-test).

Supplemental Figure 2. Western blot analysis of eIF2α and p-eIF2α with *eIF2β* overexpression

Western blotting of head extracts with anti-eIF2α (A) and anti-p-eIF2α (B) antibodies. Flies were 14 day-old. Representative blots (left) and quantitation (right) are shown. Tubulin was used as a loading control. Means ± SE, n = 6. *N.S.*, $p > 0.05$; ***$p < 0.005$ (Student's *t*-test).

Supplemental Figure 3. Lowering the eIF2β level does not affect the levels of eIF2α and p-eIF2α.

(A-C) Blotting was performed with anti-eIF2α (A) and anti-p-eIF2α (B, C) antibodies. Flies were 14 day-old. Representative blots (left) and quantitation (right) are shown. Tubulin was used as a loading control. Means ± SE, n = 6. (D) *eIF2β* gene disruption does not affect the knockdown efficiency of *milton*. *milton* mRNA levels in head extracts were quantified by qRT-PCR. Flies were 2–3 day-old. Means ± SE, n = 3. *N.S.*, $p > 0.05$; *$p < 0.05$ (Student's *t*-test).
